# Optimization of Non-Enzymatic Ultrasound-Assisted Extraction of Yellowfin Tuna Head Oil and Comparison with Enzyme-Assisted Extraction Oils

**DOI:** 10.3390/foods15142524

**Published:** 2026-07-16

**Authors:** Ruixue Cao, Yifan Ren, Zhunyao Zhu, Xiaopeng Zou, Longqi Gu, Xiangzhong Zhao

**Affiliations:** 1 School of Food Sciences and Engineering, Qilu University of Technology (Shandong Academy of Sciences), Jinan 250353, China; 13655317129@163.com (R.C.); 15288886736@163.com (Y.R.); 18366260356@163.com (Z.Z.); 18297817593@163.com (X.Z.); glq053088@163.com (L.G.); 2 Shandong Modern Marine Industry Technology Innovation Center, Tuna Processing, Qingdao 266400, China

**Keywords:** yellowfin tuna head, non-enzymatic ultrasound-assisted extraction, fish oil, response surface methodology, oxidative stability, volatile flavor, fatty acid composition

## Abstract

Yellowfin tuna heads (YFTH), a major by-product of tuna processing, are rich in lipids and long-chain n-3 polyunsaturated fatty acids. This study developed and optimized an ultrasound-assisted extraction process without exogenous protease addition, termed non-enzymatic ultrasound-assisted extraction (NE-UAE), for recovering yellowfin tuna head oil (YFTO). The term NE-UAE distinguishes this process from the enzyme-assisted comparators and does not denote a different ultrasonic mechanism. A four-factor, three-level Box–Behnken design was used to optimize nominal ultrasonic power, heat-treatment temperature, liquid-to-solid ratio, and ultrasonic treatment time. The predicted local-optimum conditions were 58.3% of the 1000 W rated power (nominal setting, 583 W), 64.0 °C, 3.0 mL/g, and 31 min, respectively, yielding a validated oil recovery of 63.92 ± 0.24%. The NE-UAE oil (NETO) was compared at the process-chain level with oils obtained by papain-, trypsin-, and Alcalase-assisted aqueous extraction. Under the tested conditions, NETO had lower acid value, initial peroxide value, and p-anisidine value, a lighter color, and lower relative abundances of putative oxidation-related electronic-nose marker signals. Polyunsaturated fatty acids comprised 38.39% of total fatty acids in NETO, including 7.43% EPA and 27.13% DHA. During accelerated storage at 60 °C for 14 d, NETO showed lower absolute peroxide values and smaller baseline-corrected increases than the comparator oils. Overall, NE-UAE provided a feasible process option for recovering YFTO with acceptable oil recovery and favorable quality-related characteristics under the specific process conditions and comparator process chains examined in this study.

## 1. Introduction

With the ongoing expansion of the global tuna capture and processing sectors, annual tuna production has reached several million tonnes. Nevertheless, the processing of canned tuna and fillets generates substantial by-products, comprising approximately 50% to 70% of fish tissues such as heads, viscera, and bones. These by-products are predominantly used for the production of fishmeal and animal feed, which have relatively low economic value [1]. Recent studies on seafood side-stream valorization have emphasized that fish-processing by-products, including heads and viscera, are rich in valuable nutrients and can be transformed into higher-value products rather than being used only for low-value applications [2]. Prior research has demonstrated that tuna heads are notably rich in lipids, containing oil on a wet basis ranging from 13% to 24%, and are abundant in n-3 long-chain polyunsaturated fatty acids (n-3 PUFAs), particularly eicosapentaenoic acid (EPA) and docosahexaenoic acid (DHA) [3,4]. These bioactive lipid constituents play significant roles in nutrition and physiological health [4,5]. Consequently, the development of efficient extraction processes to recover lipid resources from tuna-processing by-products is important for improving the value-added and sustainable utilization of aquatic resources.

Currently, marine fish oil extraction predominantly employs processes such as wet rendering, organic solvent extraction, supercritical fluid extraction, and enzyme-assisted aqueous extraction (EAAE). Wet rendering is operationally straightforward; however, the relatively elevated processing temperatures can induce oxidative degradation of PUFA-rich fish oils, thereby negatively affecting product color, oxidative status, and flavor quality [6,7]. Organic solvent extraction generally achieves high extraction efficiency, yet concerns remain regarding residual solvents and environmental safety. Supercritical fluid extraction yields high-purity lipid products but is constrained by substantial equipment investment and operational costs, limiting its feasibility for large-scale industrial application [8]. Recent work on fish oil processing has also shown that extraction and refining parameters can affect oil recovery, fatty acid composition, physicochemical quality, and volatile compound profiles, highlighting the importance of selecting appropriate processing conditions for fish oil production.

In recent years, EAAE has attracted considerable interest due to its comparatively mild and environmentally friendly processing conditions. Nonetheless, during fish oil extraction, peptides and protein hydrolysates generated through enzymatic protein hydrolysis can enhance interfacial stability within oil–water systems, leading to the formation of stable emulsions. This emulsion formation complicates subsequent centrifugal separation and diminishes the recovery of free oil [9,10]. Aitta et al. identified emulsion formation during EAAE of Baltic herring oil as a key factor affecting oil recovery efficiency and reported that reducing emulsification could improve oil recovery [11]. Furthermore, the costs associated with enzyme preparations and the need for precise process control during enzymatic hydrolysis may restrict the industrial scalability of EAAE.

Ultrasound-assisted extraction (UAE) has been extensively investigated as a green physical intensification technique for the extraction of natural products and marine bioactive compounds. The propagation of ultrasound waves through a liquid medium induces acoustic cavitation, generating localized high pressures, high-velocity microjets, and intense shear forces. These phenomena facilitate tissue disruption, enhance the release of target compounds, improve mass transfer, and increase extraction efficiency while reducing extraction time [12,13]. For lipid-rich aquatic by-products, ultrasound treatment can promote oil release under relatively mild conditions and may reduce quality deterioration associated with excessive thermal exposure. Recently, ultrasound-assisted technologies have been applied to extract fish oils from diverse sources, showing potential for improving extraction efficiency and maintaining oil quality [14,15].

Based on these considerations, this study developed an ultrasound-assisted extraction process conducted without exogenous protease addition, hereafter termed non-enzymatic ultrasound-assisted extraction (NE-UAE), for recovering yellowfin tuna head oil (YFTO). The term NE-UAE is used to distinguish the process from EAAE processes used as comparators; it does not represent an ultrasonic mechanism different from conventional UAE. The novelty of this study lies in optimizing a solvent-free, enzyme-free ultrasound-assisted process specifically for Yellowfin tuna head (YFTH) and benchmarking the complete NE-UAE process chain against practical papain-, trypsin-, and Alcalase-assisted aqueous extraction process chains using a broad set of oil-quality indicators. We hypothesized that, under the tested conditions, the shorter ultrasound-based process without exogenous protease addition could provide oil recovery comparable to the practical enzyme-assisted process chains while limiting oxidation-related quality deterioration. Six processing variables were first screened using preliminary one-factor-at-a-time experiments. Ultrasonic power, heat treatment temperature, liquid-to-solid ratio, and ultrasonic treatment time were subsequently optimized using a four-factor, three-level Box–Behnken design (BBD) based on response surface methodology (RSM). After the local optimum was validated, the effects of the four extraction process chains on oil recovery, physicochemical properties, relative volatile fingerprints, fatty acid composition, and oxidative stability were evaluated. The results may provide a process reference for the green preparation and value-added utilization of fish oil from yellowfin tuna by-products [16].

## 2. Materials and Methods

### 2.1. Materials and Chemicals

YFTH were obtained in April 2025 from Shandong Lanrun Group Co., Ltd. (Weihai, Shandong, China). The raw material comprised heads from approximately 20 yellowfin tuna collected from a single commercial processing batch. The interval between fish processing and freezing was less than 1 h. After collection, the tuna heads were immediately frozen at −80 °C and transported to the laboratory under frozen conditions.

Alcalase, trypsin, and papain were purchased from Shanghai Yuanye Bio-Technology Co., Ltd. (Shanghai, China). All other chemical reagents used in this study were of analytical grade and were purchased from Shanghai Macklin Biochemical Co., Ltd. (Shanghai, China). A 37-component fatty acid methyl ester (FAMEs) mixed standard (purity ≥ 99.4%) was purchased from Nu-Chek Prep, Inc. (Elysian, MN, USA).

### 2.2. Sample Pretreatment

Before sample preparation, all tuna heads were pooled in approximately equal mass proportions. The samples were partially thawed at 4 °C, and the gills were removed. The pooled material was then cut into small pieces using a frozen bone-cutting machine, followed by mincing and thorough homogenization using an industrial meat grinder to obtain a representative composite fish-head slurry. The homogenized slurry was divided into 500 g portions, vacuum-packed, and stored at −40 °C until further use. Independent extraction replicates were prepared using separate portions of the homogenized composite material.

### 2.3. Proximate Composition Analysis of YFTH

The proximate composition of YFTH, including moisture, crude protein, crude lipid, and ash contents, was determined according to the official methods of AOAC [17]. Moisture content was measured by drying at 105 °C to constant weight. Crude protein content was determined using the Kjeldahl method according to AOAC and Alahmad et al. [18]. Briefly, total nitrogen was measured after sulfuric acid digestion, alkaline distillation, and acid titration, and crude protein content was calculated using a nitrogen-to-protein conversion factor of 6.25. Crude lipid content was determined by Soxhlet extraction using petroleum ether as the extraction solvent. Ash content was determined gravimetrically according to AOAC, with reference to Feng et al. [19]. An accurately weighed aliquot of homogenized sample was placed in a pre-weighed crucible, carbonized until no visible smoke remained, and incinerated in a muffle furnace at 550 °C until a constant mass was obtained. After cooling in a desiccator, ash content was calculated as the mass of the remaining residue relative to the initial sample mass.

### 2.4. Extraction of YFTO Using the NE-UAE Process

YFTO was extracted using the NE-UAE process. For each extraction, 50.0 g of YFTH slurry was accurately weighed and mixed thoroughly with deionized water at the liquid-to-solid ratio specified in the experimental design.

The mixture was subjected to ultrasound treatment using an ultrasonic cell disruptor (SCIENTZ-IID, Ningbo Scientz Biotechnology Co., Ltd., Ningbo, Zhejiang, China) with a rated power of 1000 W. The probe configuration and temperature-control conditions were reported with reference to the detailed description of probe-type ultrasound-assisted lipid extraction provided by Mienis et al. [20]. A sonotrode with a tip diameter of 6 mm was immersed approximately 2 cm below the sample surface. The sample was contained in a beaker with a nominal volume of 500 mL. Ultrasonic power was expressed as a percentage of the rated instrument power; for example, a setting of 58.3% corresponded to a nominal instrument power setting of 583 W. The ultrasonic treatment time was set according to the experimental design, and ultrasound was applied in a pulsed mode of 5 s on and 5 s off.

All treatments were conducted using the same ice-bath configuration to limit temperature rise, and the sample temperature was maintained below 40 °C throughout sonication. The extraction procedure was conducted under reduced-light conditions to limit light-induced oxidation, and all samples were handled under identical conditions. Calorimetric acoustic power was not measured in this study. Therefore, the reported wattages represent nominal instrument setpoints rather than the acoustic power actually delivered to the sample, and the acoustic power density and acoustic energy density could not be determined.

Immediately after ultrasound treatment, the samples were transferred to a thermostatic water bath and extracted at the heat treatment temperature and for the duration specified in the experimental design. Following extraction, the samples were centrifuged at 10,000× *g* for 20 min at 20 °C. The upper free-oil layer was collected, weighed (*m*_1_), and stored at −40 °C until further analysis. Oil recovery (%) was calculated according to Equation (1):

(1) $$Y(\%)\;=\frac{\;m_{1}}{\;(m_{0}\;\times \;Lc)}\;\;\times \;100$$

where *Y* is the oil recovery (%), *m*_1_ is the mass of free oil obtained after centrifugation (g), *m*_0_ is the mass of YFTH slurry introduced into the extraction system (g), and *Lc* is the crude lipid content of YFTH on a wet basis, which was determined to be 14.90% (*w*/*w*) based on the proximate composition analysis. For calculation, *Lc* was expressed as 0.1490. Oil recovery represents the proportion of total lipids in the raw material recovered as free oil.

### 2.5. Optimization of the Extraction Process

#### 2.5.1. Preliminary One-Factor-at-a-Time Experiments

Preliminary one-factor-at-a-time (OFAT) experiments were conducted to screen six processing variables affecting oil recovery and to determine suitable ranges for subsequent response surface optimization. The initial parameter ranges were selected with reference to previous work on ultrasound-assisted fish oil extraction, particularly the study by Mai et al. [21], and were further refined based on preliminary experiments. For each experimental series, only the variable under investigation was changed, while the remaining variables were maintained at the following reference conditions, as applicable: ultrasonic power, 60% of the 1000 W rated power; heat treatment temperature, 65 °C; liquid-to-solid ratio, 3 mL/g; ultrasonic treatment time, 30 min; minced YFTH particle size, 2 mm; and heat treatment time, 20 min.

The levels evaluated were 20%, 40%, 60%, 80%, and 100% for ultrasonic power; 45, 55, 65, 75, and 85 °C for heat treatment temperature; 1, 2, 3, 4, and 5 mL/g for liquid-to-solid ratio; 10, 20, 30, 40, and 50 min for ultrasonic treatment time; 2, 4, 6, 8, and 10 mm for minced YFTH particle size; and 20, 30, 40, 50, and 60 min for heat treatment time. Each experimental condition was performed in triplicate, and oil recovery was used as the sole response variable during the screening stage.

#### 2.5.2. Box–Behnken Design and Response Surface Methodology

A four-factor, three-level BBD was used to optimize oil recovery. The independent variables were ultrasonic power (*X*_1_), heat treatment temperature (*X*_2_), liquid-to-solid ratio (*X*_3_), and ultrasonic treatment time (X_4_). The coded and actual levels were 40%, 60%, and 80% of rated power for *X*_1_; 55, 65, and 75 °C for *X*_2_; 2, 3, and 4 mL/g for *X*_3_; and 20, 30, and 40 min for *X*_4_, respectively (Table S1). These ranges were centered on the regions showing relatively high oil recovery in the OFAT experiments. The design comprised 29 experimental runs, including 24 factorial runs and five center-point replicates (Table S2). Although ultrasonic power and ultrasonic treatment time both contribute to nominal ultrasound energy input, they were retained as separately controllable factors representing treatment intensity and treatment duration, respectively. Their combined effect was evaluated through the *X*_1_*X*_4_ interaction term. The resulting data were fitted to a second-order polynomial regression model, as expressed in Equation (2):

(2) $$Y\;=\;\beta_{0}+\;\sum_{\left\{i=1\right\}}^{4}\beta_{i}X_{i}+\;\sum_{\left\{i=1\right\}}^{4}\beta_{\left\{ii\right\}X_{i}^{2}}+\;\sum_{\left\{i=1\right\}}^{3}\sum_{\left\{j=i+1\right\}}^{4}\beta_{\left\{ij\right\}X_{i}X_{j}}$$

where *Y* denotes the predicted oil recovery; *β*_0_ is the intercept term; *β_i_*, *β_ii_*, and *β_ij_* represent the linear, quadratic, and interaction coefficients, respectively; and *X_i_* and *X_j_* correspond to the coded independent variables.

### 2.6. Preparation of Enzyme-Assisted Extraction Oil Samples

To evaluate the effects of different extraction processes on YFTO quality, oil samples were prepared by enzyme-assisted extraction using papain, trypsin, and Alcalase. The hydrolysis conditions were selected based on previous studies and preliminary experiments [3,14]. YFTH slurry was mixed with deionized water at a liquid-to-solid ratio of 3 mL/g, and the pH was adjusted to the target value for each enzyme using 1 M HCl or 1 M NaOH. The enzymatic reactions were conducted under the following conditions: papain, pH 7.0 and 55 °C; trypsin, pH 8.0 and 40 °C; and Alcalase, pH 8.0 and 55 °C.

The enzyme dosage was set at 6000 U/g crude protein for all groups to provide a common practical comparison basis, and hydrolysis was performed in a thermostatic water bath for 2 h. These enzyme-assisted extraction conditions were used as practical comparative conditions rather than individually optimized conditions for each enzyme. However, the same nominal enzyme dosage did not necessarily ensure equivalent catalytic activity or hydrolysis extent among the three enzymes. After hydrolysis, the samples were heated at 95 °C for 15 min to inactivate the enzymes, followed by centrifugation at 10,000× *g* for 20 min. The upper free-oil layer was collected and designated as papain-assisted extraction oil (PTO), trypsin-assisted extraction oil (TTO), and Alcalase-assisted extraction oil (ATO), respectively. The oil samples were stored at −40 °C until further analysis. Because these treatments differed from NE-UAE in pH adjustment, processing duration, ultrasound exposure, and thermal history, including the 95 °C enzyme-inactivation step, the comparisons in this study represent complete process-chain comparisons rather than isolated comparisons of enzyme addition or extraction principle.

### 2.7. Quality Assessment of Oil Samples

#### 2.7.1. Basic Physicochemical Properties

Moisture and volatiles, acid value (AV), peroxide value (POV), p-anisidine value (p-AnV), unsaponifiable matter, iodine value (IV), and insoluble impurities of oil samples obtained using different extraction processes were determined according to the American Oil Chemists’ Society (AOCS) official methods [22].

#### 2.7.2. Color Measurement

Oil color was measured in transmission mode using a colorimeter (LICO 690, Hach, Loveland, CO, USA). The instrument was operated with a D65 standard illuminant and a 10° observer angle and calibrated using distilled water as a blank. Samples were placed in 1 cm quartz cuvettes, and L*, a*, b*, chroma (C*), hue angle (h°), and yellowness index (YI) were recorded.

#### 2.7.3. Volatile Flavor Analysis

Headspace volatile fingerprints of the oil samples were obtained using a flash gas chromatography-based electronic nose system (GC-E-nose; Heracles NEO 300G, Alpha MOS, Toulouse, France) [23,24]. Oil samples (0.50 g) were placed in 20 mL headspace vials, equilibrated at 80 °C for 20 min, and automatically injected. Volatile compounds were enriched in a trap, separated on dual GC columns, and detected using a flame ionization detector (FID). Data were processed using AlphaSoft software (version 2024, Alpha MOS, Toulouse, France). Retention indices (RIs) were calculated using a homologous series of n-alkanes, and volatile compounds were tentatively identified using the AroChemBase database. Peak areas were used for analysis, and relative standard deviation (RSD) was used to evaluate repeatability and instrumental stability. Principal component analysis (PCA) and clustered heatmap analysis were used to compare volatile flavor profiles among oil samples.

### 2.8. Fatty Acid Composition Analysis

Fatty acid composition was determined using gas chromatography–mass spectrometry (GC–MS), with appropriate modifications to a previously reported method [25]. Lipids were extracted from oil samples using a chloroform–dichloromethane solvent system and methylated with methanol containing 5% sulfuric acid at 80 °C for 2 h. After the reaction, FAMEs were extracted with n-hexane. After centrifugation, the upper organic phase was collected, made up to volume with isooctane, and used for GC–MS analysis.

FAMEs were analyzed using a GC–MS system equipped with a single quadrupole mass spectrometer (ISQ 7000, Thermo Fisher Scientific, Waltham, MA, USA). Separation was performed on an Agilent HP-88 capillary column (100 m × 0.25 mm × 0.20 μm). The injection volume was 1 μL, the split ratio was 20:1, and high-purity helium was used as the carrier gas at a flow rate of 1.0 mL/min. The oven temperature program was as follows: 40 °C for 3 min; increased to 160 °C at 12 °C/min; increased to 215 °C at 1.2 °C/min and held for 2 min; and finally increased to 280 °C at 5 °C/min and held for 10 min. The mass spectrometer was operated in electron ionization (EI) mode at 70 eV, with the ion source and transfer line temperatures set at 280 °C. Data were acquired in full-scan mode over an *m*/*z* range of 35–550. Fatty acids were identified by comparing retention times and mass spectra with those of the FAMEs mixed standard. Relative proportions were calculated by peak area normalization and expressed as percentages of total fatty acids.

### 2.9. Accelerated Oxidative Storage Test

Oil samples (10 g) were sealed in amber glass bottles and stored at 60 °C in the dark. Samples were collected at predetermined intervals for POV determination. NETO, PTO, TTO, and ATO were stored under identical conditions. To account for differences in the initial POV among the oil samples, baseline-corrected changes were calculated as ΔPOV_t_ = POV_t_ − POV_0_, where POV_t_ and POV_0_ represent the peroxide values at storage time t and day 0, respectively.

### 2.10. Statistical Analysis

All experimental measurements were performed using three independent extraction replicates, and the results are presented as mean ± standard deviation (SD) (*n* = 3). Statistical analyses were performed using one-way analysis of variance (ANOVA) with IBM SPSS Statistics software version 26.0. Duncan’s multiple range test was used for post hoc comparison among groups, and differences were considered statistically significant at *p* < 0.05. RSM analysis and Box–Behnken design optimization were performed using Design-Expert software version 13.0. Principal component analysis (PCA), heatmap visualization, and other graphical analyses were performed using Origin (version 2024, OriginLab Corporation, Northampton, MA, USA) and R software (version 4.4.1, R Foundation for Statistical Computing, Vienna, Austria). For heatmap analysis, peak-area data were normalized using Z-score transformation before visualization.

## 3. Results and Discussion

### 3.1. Proximate Composition of YFTH

The proximate composition of YFTH is shown in Table 1. The moisture, crude protein, crude lipid, and ash contents were 59.15 ± 0.51, 15.32 ± 0.15, 14.90 ± 0.21, and 8.90 ± 0.24 g/100 g, respectively. The relatively high crude lipid content indicates that YFTH has potential as a raw material for fish oil extraction. This result is consistent with de Oliveira et al., who reported that oil recovered from yellowfin tuna-processing by-products could serve as a potential source for fish oil utilization [26]. In addition, the relatively high crude protein and ash contents in YFTH may be related to the presence of muscle, connective tissue, and bone components in fish heads. Li et al. also reported that fish head by-products contain certain proportions of protein, lipid, and mineral components [27].

From the perspective of oil extraction, the protein matrix and bone tissues may limit lipid release and affect the separation and recovery of free oil. Therefore, suitable structural disruption or mass-transfer intensification strategies are needed in subsequent extraction processes to promote oil release. Overall, YFTH contains a relatively high crude lipid content and a rich proximate composition, providing a suitable raw material basis for the preparation of high-quality YFTO.

### 3.2. Preliminary One-Factor-at-a-Time Screening

**Figure 1:**
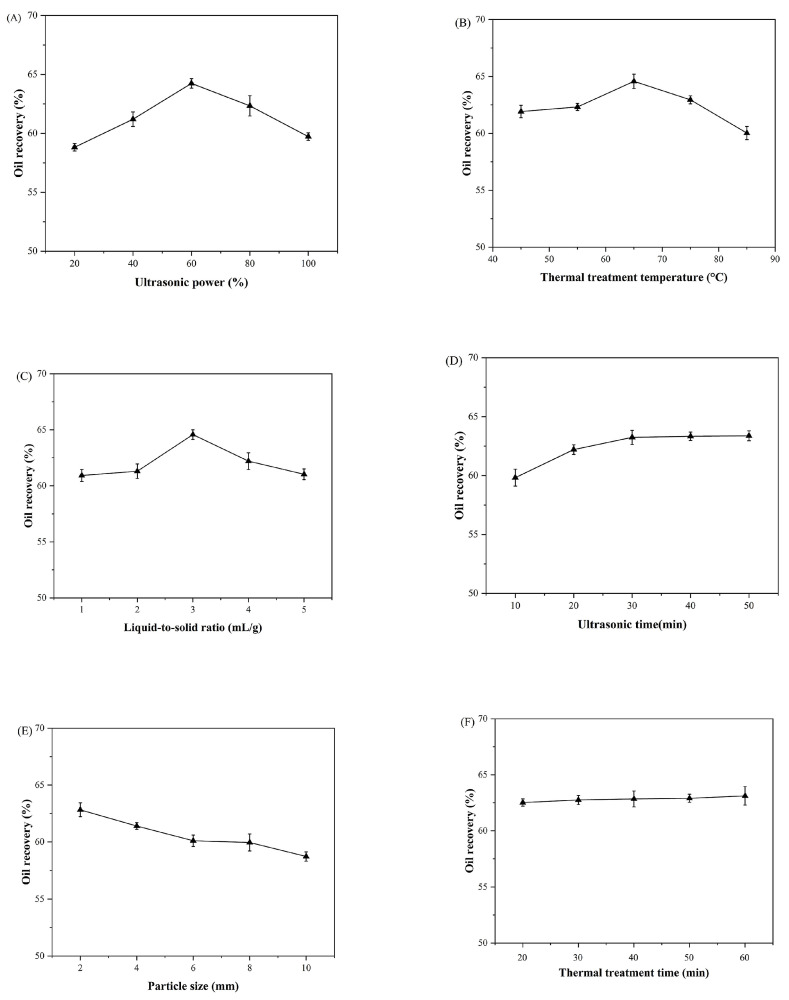
Effects of processing parameters on oil recovery in the NE-UAE process. Note: (**A**) ultrasonic power; (**B**) heat treatment temperature; (**C**) liquid-to-solid ratio; (**D**) ultrasonic treatment time; (**E**) minced YFTH particle size; and (**F**) heat treatment time. Values are expressed as mean ± SD (*n* = 3).

To screen the processing variables and identify suitable ranges for response surface optimization, ultrasonic power, heat treatment temperature, liquid-to-solid ratio, ultrasonic treatment time, minced YFTH particle size, and heat treatment time were evaluated using the OFAT approach (Figure 1). In each experimental series, only one variable was changed, while the remaining variables were maintained at the reference conditions described in Section 2.5.1. Oil recovery increased as ultrasonic power increased from 20% to 60% and then decreased at 80% and 100%. Heat treatment temperature and liquid-to-solid ratio showed similar increasing–decreasing trends, with relatively high oil recovery observed at approximately 65 °C and 3 mL/g, respectively. Oil recovery increased as ultrasonic treatment time increased from 10 to 30 min and then approached a plateau. These trends indicate that moderate ultrasonic intensity and treatment duration may enhance cavitation, tissue disruption, and mass transfer, whereas further increases did not provide additional improvement in free oil recovery [12,28].

Increasing minced YFTH particle size from 2 to 10 mm gradually reduced oil recovery, whereas extending heat treatment time from 20 to 60 min produced only small changes. Accordingly, minced YFTH particle size and heat treatment time were fixed at 2 mm and 20 min, respectively. Ultrasonic power (*X*_1_), heat treatment temperature (*X*_2_), liquid-to-solid ratio (*X*_3_), and ultrasonic treatment time (*X*_4_) were selected as the four independent variables for subsequent BBD optimization. The BBD data shown in Table S2 represent the subsequent multivariable optimization stage and are separate from the OFAT screening experiments presented in Figure 1. Because particle size and heat treatment time were fixed on the basis of oil recovery rather than oxidation or flavor indices, their possible effects on oil quality were not optimized and should be considered a limitation of the yield-oriented design.

### 3.3. BBD Optimization and Model Analysis

#### 3.3.1. Model Fitting and ANOVA

To optimize the effects of ultrasonic power (*X*_1_), heat treatment temperature (*X*_2_), liquid-to-solid ratio (*X*_3_), and ultrasonic treatment time (*X*_4_) on oil recovery, a BBD was used, and 29 experimental runs were performed. The experimental design and results are shown in Table S2. The experimental oil recovery values ranged from 53.24% to 64.61%, while the predicted values ranged from 53.11% to 64.19%, showing good agreement between the experimental and predicted values. Based on the experimental data, a second-order polynomial regression model for oil recovery (*Y*) was established as follows:

(3) $$\begin{array}{c} Y\;=\;64.19\;-\;0.5817X_{1}-\;1.41X_{2}+\;0.1358X_{3}+\;0.2358X_{4}-\;0.2350X_{1}X_{2}+\;0.0950X_{1}X_{3}\\ +\;0.600X_{1}X_{4}-\;0.0850X_{2}X_{3}-\;0.5150X_{2}X_{4}-\;0.1475X_{3}X_{4}-\;3.29X_{1}^{2}-\;5.56X_{2}^{2}-\;2.19X_{3}^{2}-\;2.15X_{4}^{2} \end{array}$$

where *Y* is the predicted oil recovery (%), and *X*_1_, *X*_2_, *X*_3_, and *X*_4_ represent ultrasonic power, heat treatment temperature, liquid-to-solid ratio, and ultrasonic treatment time, respectively.

The ANOVA results are shown in Table S3. The regression model was highly significant (F = 175.47, *p* < 0.0001), while the lack of fit was not significant (*p* = 0.3775), indicating that the fitted quadratic model adequately described the experimental data. The R^2^ value was 0.9943, indicating that 99.43% of the variation in oil recovery could be explained by the model. The adjusted R^2^ (0.9887) and predicted R^2^ (0.9724) were close, suggesting good model fit and predictive ability. The Adeq Precision value was 46.33, which was higher than the recommended value of 4, indicating an adequate signal for process optimization and response prediction. The C.V. was 0.57%, indicating good repeatability of the experimental results.

#### 3.3.2. Response Surface and Interaction Analysis

**Figure 2:**
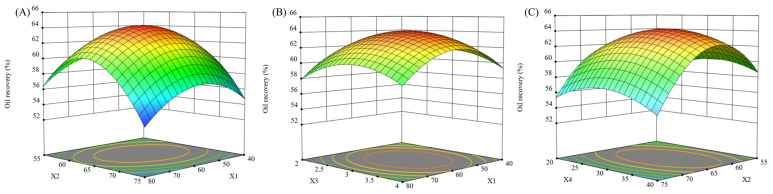
Response surface plots showing the effects of parameter interactions on oil recovery in the NE-UAE process. Note: (**A**) ultrasonic power (*X*_1_) and heat treatment temperature (*X*_2_); (**B**) ultrasonic power (*X*_1_) and liquid-to-solid ratio (*X*_3_); (**C**) heat treatment temperature(*X***_2_**) and ultrasonic treatment time (*X*_4_).

The three-dimensional response surface plots (Figure 2) show the effects of different parameter combinations on oil recovery. According to the ANOVA results (Table S3), the quadratic terms of the four factors selected for BBD optimization (*X*_1_^2^, *X*_2_^2^, *X*_3_^2^, and *X*_4_^2^) were all highly significant (*p* < 0.0001), indicating nonlinear effects of these factors on oil recovery within the investigated ranges. As shown in Figure 2A, ultrasonic power and heat treatment temperature jointly affected oil recovery, with higher oil recovery obtained when both factors were at moderate levels. However, their interaction term (*X*_1_*X*_2_) was not significant (*p* = 0.1793), suggesting that the response surface variation was mainly associated with their individual effects rather than a significant synergistic effect. Moderate ultrasonic power may enhance cavitation, tissue disruption, and lipid release, while an appropriate heat treatment temperature may reduce oil viscosity and improve mass transfer, thereby increasing oil recovery [12,13].

Figure 2B shows that oil recovery reached a relatively high level near the central region when ultrasonic power and liquid-to-solid ratio were varied together. However, the interaction between these two factors (*X*_1_*X*_3_) was not significant (*p* = 0.5768). This indicates that, within the tested range, the liquid-to-solid ratio mainly affected oil release by improving mass transfer conditions and the ultrasound energy transfer environment, rather than by exerting a clear interaction with ultrasonic power.

Ultrasonic power and ultrasonic treatment time are physically related because both contribute to the nominal ultrasound energy input. However, the *X*_1_*X*_4_ interaction was not statistically significant within the investigated ranges (F = 0.1302, *p* = 0.7236; Table S3). This result indicates that the effect of ultrasonic power on oil recovery did not vary significantly with treatment time between 20 and 40 min at power settings of 40–80%. The absence of a significant interaction should not be interpreted as physical independence between power and duration. Because *X*_1_*X*_4_ was not significant, this factor pair was not selected for presentation among the representative response surface plots in Figure 2.

In contrast, heat treatment temperature and ultrasonic treatment time showed a significant interaction effect (*X*_2_*X*_4_, *p* = 0.0079) (Figure 2C). Higher oil recovery was obtained under moderate heat treatment temperature and ultrasonic treatment time, whereas further increases in temperature or treatment time reduced oil recovery. This suggests that appropriate heat treatment combined with ultrasound may promote tissue disruption and lipid migration, while excessive energy input may reduce separation efficiency and limit further free oil recovery.

#### 3.3.3. Process Optimization and Model Validation

Numerical optimization based on the quadratic regression model gave the theoretical optimal NE-UAE conditions as follows: ultrasonic power, 58.3%; heat treatment temperature, 63.7 °C; liquid-to-solid ratio, 3.0 mL/g; and ultrasonic treatment time, 30.7 min. Under these conditions, the predicted oil recovery was 64.32%. Considering the control accuracy of the equipment, the validation conditions were adjusted to an ultrasonic power of 58.3%, heat treatment temperature of 64.0 °C, liquid-to-solid ratio of 3.0 mL/g, and ultrasonic treatment time of 31 min. Three independent validation experiments were then performed.

The oil recovery values obtained from the validation experiments were 63.80%, 64.20%, and 63.76%, with an average value of 63.92 ± 0.24%. The absolute and relative differences between the experimental mean and the model-predicted value were 0.40 percentage points and 0.62%, respectively. This agreement confirms that the model successfully identified and reproduced the local optimum under the selected validation conditions. However, because validation was conducted only near the mathematical optimum, it does not constitute an independent assessment of predictive performance across the entire design space. Therefore, the model should be interpreted primarily as an optimization tool within the investigated factor ranges. NETO samples for subsequent quality comparisons were prepared under the validated local-optimum conditions.

### 3.4. Comparison of Oil Recovery Among Different Extraction Processes

Different extraction process chains significantly affected the oil recovery of YFTO (Table 2). The comparison was designed to benchmark the complete NE-UAE process against three practical enzyme-assisted aqueous extraction process chains; it was not intended to isolate the effect of enzyme addition from differences in pH adjustment, processing duration, ultrasound treatment, and thermal history. Under the process conditions used in this study, the oil recovery values followed the order ATO (67.72 ± 0.41%) > NETO (63.92 ± 0.24%) > PTO (62.69 ± 0.38%) > TTO (56.48 ± 0.52%).

ATO showed the highest oil recovery, which may be related to the strong proteolytic activity of Alcalase under the selected hydrolysis conditions. Extensive hydrolysis of protein networks and connective tissues may facilitate the release of embedded lipids, thereby improving free oil recovery. In contrast, the lower oil recovery observed in PTO and TTO may be associated with differences in enzyme specificity and hydrolysis efficiency.

NETO achieved an oil recovery of 63.92 ± 0.24%, which was lower than that of ATO but higher than those of PTO and TTO. This result suggests that the NE-UAE process may promote tissue disruption and lipid release under the tested conditions. Cavitation, microjets, and mechanical shear generated by ultrasound treatment can enhance structural disruption and mass transfer, thereby facilitating oil release from the tissue matrix, which is consistent with recent studies on ultrasound-assisted fish oil extraction and ultrasound-assisted lipid extraction mechanisms [15,29].

In addition, soluble protein hydrolysates and peptides generated during EAAE may exhibit interfacial activity and promote the formation of an emulsion layer, causing part of the oil to remain in the emulsified phase and affecting subsequent centrifugal separation and free oil recovery [30]. Compared with the enzyme-assisted extraction processes, the NE-UAE process did not involve enzymatic hydrolysis, which may have reduced the formation of large amounts of protein hydrolysates and facilitated oil–water separation. However, the comparison in this study was conducted at the process level. The NE-UAE and enzyme-assisted extraction treatments differed not only in enzyme addition but also in ultrasound treatment, pH, processing duration, and thermal history. Therefore, the observed differences in oil recovery should be interpreted as the combined effects of the extraction process chains rather than the effect of enzyme absence alone.

Overall, under the tested conditions, NE-UAE achieved an oil recovery close to that of ATO without using exogenous enzymes, suggesting that it may provide a feasible process option for recovering YFTO from YFTH.

### 3.5. Physicochemical Properties

#### 3.5.1. Effects of Extraction Processes on the Basic Physicochemical Properties of YFTO

For AV, NETO showed the lowest value of 0.77 ± 0.07 mg KOH/g, compared with PTO (1.51 ± 0.06 mg KOH/g), TTO (2.11 ± 0.06 mg KOH/g), and ATO (1.88 ± 0.11 mg KOH/g), and met the refined-fish-oil limit shown in Table 3. A lower AV indicates less accumulation of free fatty acids. The differences observed here may reflect the combined effects of the complete process chains, including aqueous exposure, pH adjustment, processing duration, proteolysis, ultrasound treatment, and thermal history, rather than enzyme addition alone. Ultrasound-assisted extraction has been reported to enhance tissue disruption and mass transfer and may reduce processing duration and thermal exposure, which could help limit lipid-quality deterioration under suitable conditions [30].

For POV, NETO showed the lowest initial value of 1.85 ± 0.13 mmol/kg, compared with PTO (6.43 ± 0.16 mmol/kg), TTO (10.25 ± 0.16 mmol/kg), and ATO (16.68 ± 0.19 mmol/kg). The higher values in the enzyme-assisted oils cannot be attributed solely to enzymatic hydrolysis. In addition to prolonged aqueous hydrolysis, these process chains included pH adjustment and heating at 95 °C for 15 min for enzyme inactivation, which may have contributed substantially to oxidation. Emulsion formation and increased oil–water interfacial area may also have affected oil separation and oxidation [14]. Thus, the observed POV differences should be interpreted as combined process-chain effects.

IV reflects the overall degree of unsaturation and double-bond extraction processes of oils. NETO showed an IV of 184 ± 2.16 g/100 g, compared with 154 ± 1.80, 164 ± 2.46, and 134 ± 1.37 g/100 g for PTO, TTO, and ATO, respectively. These values indicate differences in the relative unsaturation of the oils obtained under the tested process conditions; however, they do not by themselves demonstrate absolute retention or recovery of individual PUFAs. Previous reviews have suggested that ultrasound-based green extraction processes may reduce thermal exposure and processing intensity, thereby helping limit the degradation of highly unsaturated fatty acids under appropriate operating conditions [30].

By comparison, moisture and volatiles (0.07–0.10%), unsaponifiable matter (0.60–0.88%), and insoluble impurities (0.04–0.10%) showed relatively small differences among the treatment groups and remained at low levels, indicating that the extraction processes had limited effects on these indices.

Overall, the NETO process reduced lipid hydrolysis and primary oxidation while maintaining a higher degree of unsaturation, showing better overall oil quality than the three enzyme-assisted extraction processes.

#### 3.5.2. Effects of Extraction Processes on the Color Properties of YFTO

**Figure 3:**
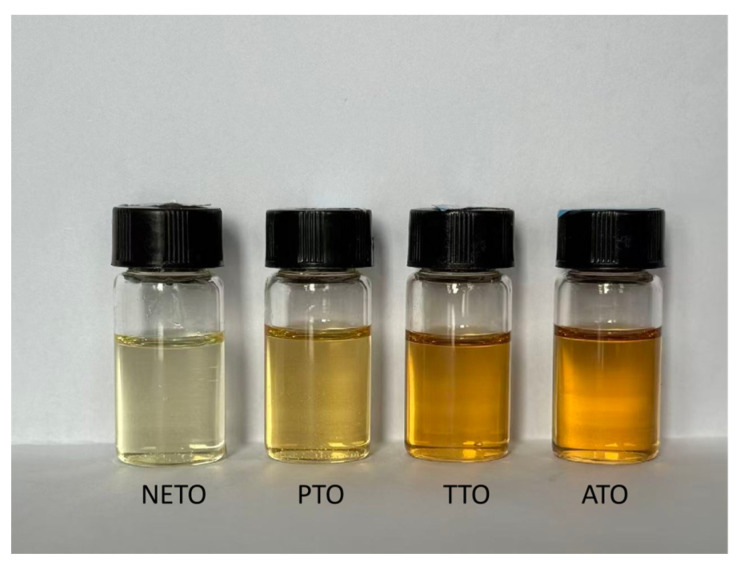
Appearance of YFTO obtained using different extraction processes. Note: From left to right, the samples are NETO, PTO, TTO, and ATO.

Figure 3 and Table 4 show clear differences in the color properties of YFTO obtained using different extraction processes. NETO had the highest L* and h° values, while its a* value was close to zero and slightly negative. Its YI was also lower than those of TTO and ATO, indicating a clearer and lighter yellow appearance. PTO, TTO, and ATO showed lower L* values and higher a*, C*, and YI values, suggesting darker colors and more pronounced reddish-yellow tones. The high YI values of TTO and ATO further indicate a stronger yellow to brownish-yellow appearance.

The differences in color may be associated with the effects of extraction processes on tissue disruption, migration of accompanying components, and lipid oxidation. Protease treatment can degrade protein networks and connective tissues in fish heads, promoting the release of bound lipids and possibly facilitating the migration of pigments, protein degradation products, and other cellular components into the oil phase. Mbatia et al. reported that enzymatic treatment promoted oil release from fish heads through tissue degradation [8], which may partly explain the color changes observed in the enzyme-assisted extraction groups.

The prolonged hydrolysis and high-temperature enzyme inactivation steps used in enzyme-assisted extraction may also promote lipid oxidation and reactions between oxidation products and protein degradation products, leading to the formation of colored oxidation products or browning-related compounds. Bao et al. reported that dicarbonyl compounds generated from lipid oxidation can react with food proteins and participate in protein oxidation and browning-related product formation [31]. Therefore, the lower L* values and higher a* and YI values of PTO, TTO, and ATO may be related to color deepening induced by enzymatic hydrolysis and heat treatment.

The NETO process mainly relies on ultrasound-induced cavitation and mechanical shear to promote oil release, without exogenous enzymatic hydrolysis or high-temperature enzyme inactivation. This may reduce the formation of protein hydrolysates and heat-induced color deterioration. Recent studies on ultrasound-assisted fish oil extraction have also shown that appropriate ultrasound treatment can improve extraction efficiency while maintaining good oil quality [15]. Based on the color parameters in Table 4, the NE-UAE process helped maintain higher lightness and a lower yellowness index of YFTO, thereby improving the appearance quality of the extracted oil.

#### 3.5.3. Relative Oxidation-Marker Fingerprints and Putative Oxidation-Related Signals

##### PCA of Relative Oxidation-Marker Fingerprints

PCA was performed using electronic-nose peak-area data to compare the relative oxidation-marker fingerprints of YFTO obtained using the four extraction process chains. PCA reduces the dimensionality of complex signal datasets and facilitates visualization of overall differences among sample patterns. This approach has been applied to volatile fingerprint analysis of aquatic products and processed fish products [32,33].

**Figure 4:**
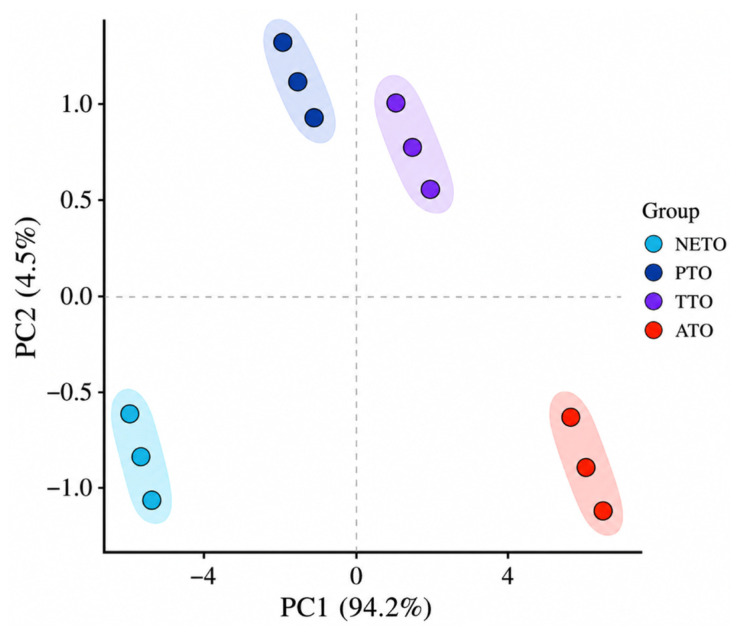
PCA score plot of relative electronic-nose volatile fingerprints of YFTO obtained using different extraction process chains. Note: PC1 and PC2 represent the first and second principal components, respectively. NETO, non-enzymatic ultrasound-assisted extraction oil; PTO, papain-assisted extraction oil; TTO, trypsin-assisted extraction oil; ATO, Alcalase-assisted extraction oil.

As shown in Figure 4, PC1 and PC2 explained 94.2% and 4.5% of the total variance, respectively, accounting for a cumulative contribution of 98.7%. Thus, the first two principal components summarized most of the variation in the relative oxidation-marker fingerprints. The three independent extraction replicates within each treatment group clustered closely in the score plot, indicating good within-treatment consistency of the fingerprint patterns [34].

NETO, PTO, TTO, and ATO were distributed in distinct regions of the PCA score plot without obvious overlap, indicating differences in their overall relative oxidation-marker fingerprints. NETO was separated from the three enzyme-assisted extraction oils mainly along PC1, suggesting that the complete NE-UAE process chain produced a different overall signal pattern from those of the enzyme-assisted process chains. Similar sample discrimination based on volatile fingerprints has been reported for processed tuna products and processed fish oils [33,35].

Because the individual electronic-nose signals were tentatively assigned only by retention-index matching with the database and were not confirmed by GC–MS or calibrated using authentic standards, the results represent relative fingerprint discrimination of putative oxidation-related marker signals rather than confirmed identification, absolute quantification, or comprehensive characterization of the volatile fraction. The putative oxidation-related signals associated with the observed sample separation were further examined using clustered heatmap analysis.

##### Relative-Abundance Heatmap Analysis of Putative Oxidation-Related Marker Signals

**Figure 5:**
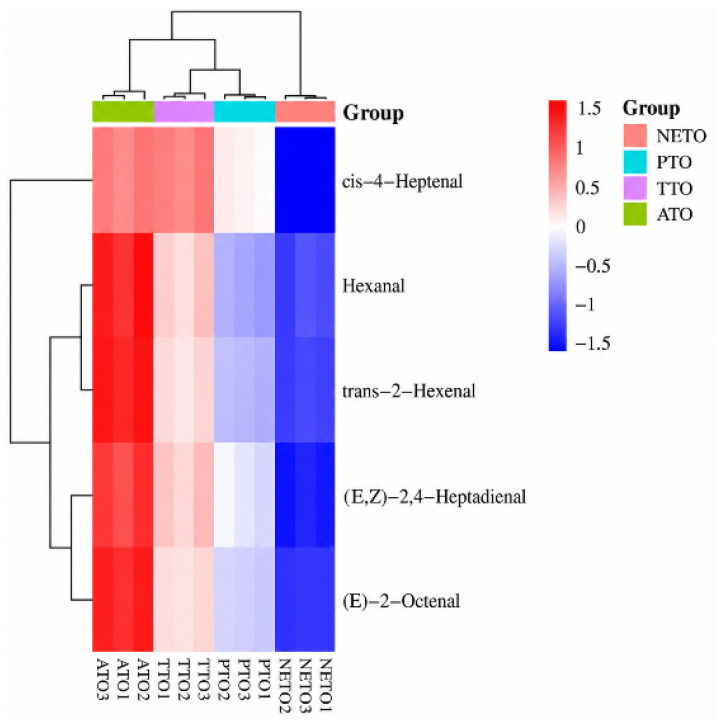
Clustered heatmap of putative oxidation-related aldehyde marker signals in YFTO obtained using different extraction process chains. Note: Columns represent samples and replicates, and rows represent five signals tentatively assigned by retention-index database matching. The heatmap was generated from Z-score-normalized peak areas. Red indicates higher relative signal abundance, and blue indicates lower relative signal abundance. NETO, non-enzymatic ultrasound-assisted extraction oil; PTO, papain-assisted extraction oil; TTO, trypsin-assisted extraction oil; ATO, Alcalase-assisted extraction oil.

To further compare the relative oxidation-marker fingerprints of YFTO obtained using different extraction process chains, five electronic-nose signals tentatively assigned to *cis-*4-heptenal, hexanal, *trans*-2-hexenal, (*E*, *Z*)-2,4-heptadienal, and (*E*)-2-octenal were selected on the basis of retention-index matching with the database. A clustered heatmap was constructed using *Z*-score-normalized peak areas to visualize differences in their relative signal abundances among the oil samples (Figure 5).

As shown in Figure 5, the oils obtained using the four extraction process chains exhibited distinct clustering patterns based on the relative abundances of these putative oxidation-related marker signals. NETO generally showed lower relative signal abundances, whereas ATO showed higher relative abundances, with PTO and TTO displaying intermediate patterns. These differences reflect relative electronic-nose fingerprint patterns and do not represent absolute concentrations of the corresponding volatile compounds. Because the tentatively assigned aldehydes have been associated with grassy, oxidized, fishy, or rancid odor attributes, the lower relative signal abundances observed in NETO suggest a lower relative expression of putative oxidation-related off-odor markers under the tested conditions.

Hexanal and *trans*-2-hexenal have been reported as volatile products associated with the oxidation of unsaturated fatty acids and with grassy or oxidized odor characteristics. Signals tentatively assigned to *cis*-4-heptenal and (*E*, *Z*)-2,4-heptadienal may be associated with the oxidative degradation of long-chain n-3 PUFAs, including EPA and DHA, and have been reported as potential markers of fishy odor and oxidative flavor deterioration in fish oil [36]. Similarly, (*E*)-2-octenal has been associated with lipid oxidation and has been proposed as a volatile indicator of oxidative deterioration in oils [37]. Therefore, the lower relative abundances of these putative marker signals in NETO may indicate less pronounced oxidation-related volatile fingerprint characteristics under the tested process conditions.

The relative electronic-nose fingerprint patterns were generally consistent with the physicochemical and color results. NETO showed the lowest initial POV and a lighter color, while also exhibiting lower relative abundances of the selected putative oxidation-related marker signals. Although ATO achieved the highest oil recovery, it showed higher relative abundances of several putative oxidation-related signals. These differences may reflect the combined effects of the complete extraction process chains, including aqueous hydrolysis, pH adjustment, processing duration, emulsion formation, and the subsequent high-temperature enzyme-inactivation step. Overall, the electronic-nose results provide comparative relative oxidation-marker fingerprints among the oil samples rather than confirmed identification, absolute quantification, or comprehensive characterization of their volatile composition.

### 3.6. Fatty Acid Composition Analysis

**Figure 6:**
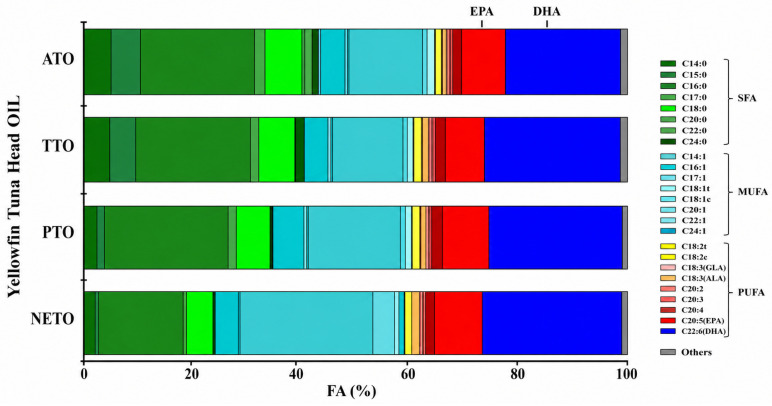
Relative fatty acid composition of YFTO obtained using different extraction process chains. Note: The stacked bars represent peak-area-normalized fatty acid proportions (% of total identified fatty acids), not absolute concentrations or recoveries. EPA and DHA are highlighted. SFA, MUFA, and PUFA represent saturated, monounsaturated, and polyunsaturated fatty acids, respectively. NETO, non-enzymatic ultrasound-assisted extraction oil; PTO, papain-assisted extraction oil; TTO, trypsin-assisted extraction oil; ATO, Alcalase-assisted extraction oil.

To evaluate the effects of different extraction processes on the fatty acid composition of YFTO, NETO, PTO, TTO, and ATO were analyzed (Table 5), and a stacked bar chart of fatty acid composition was generated (Figure 6).

As shown in Table 5 and Figure 6, palmitic acid (C16:0), oleic acid (C18:1n9c), EPA, and DHA were the major fatty acids in all YFTO samples. This fatty acid profile is consistent with previous studies showing that fish-processing by-products can serve as important sources of omega-3 fatty acids [38]. The extraction process chains were associated with differences in the relative proportions of major fatty acids and fatty acid classes. NETO showed relative proportions of 26.55% ΣSFA, 35.00% ΣMUFA, and 38.39% ΣPUFA. Because these values were calculated by peak-area normalization, they describe relative fatty acid composition only and do not provide absolute fatty acid concentrations or absolute recovery from the raw material.

The relative proportions of EPA and DHA in NETO were 7.43% and 27.13%, respectively. These percentages were higher than the corresponding proportions in PTO, TTO, and ATO under the tested conditions. Comparable increases in the relative proportions of omega-3 fatty acids and PUFAs after ultrasound-assisted extraction have also been reported for oil recovered from Atlantic bonito by-products [39]. However, a higher normalized percentage may partly reflect lower relative proportions of other fatty acids and cannot be interpreted as higher absolute nutritional recovery or absolute preservation of EPA and DHA. Determination of absolute EPA and DHA recovery would require calibrated concentration measurements combined with total oil-recovery data.

Differences in relative fatty acid composition may have arisen from the combined effects of the complete extraction process chains. The enzyme-assisted treatments included pH adjustment, 2 h of aqueous hydrolysis, enzyme-specific reaction temperatures, and subsequent heating at 95 °C for 15 min, whereas NE-UAE involved pulsed ultrasound and a shorter moderate-temperature treatment. Previous studies have shown that the fatty acid composition and quality of fish oil obtained after enzymatic hydrolysis can be influenced by pretreatment conditions, processing history, storage, and lipid oxidation [40]. Therefore, the observed relative compositional differences cannot be attributed solely to enzymatic hydrolysis or interpreted as direct evidence of selective PUFA retention.

### 3.7. Effects of Different Extraction Processes on the Oxidative Stability of YFTO

**Figure 7:**
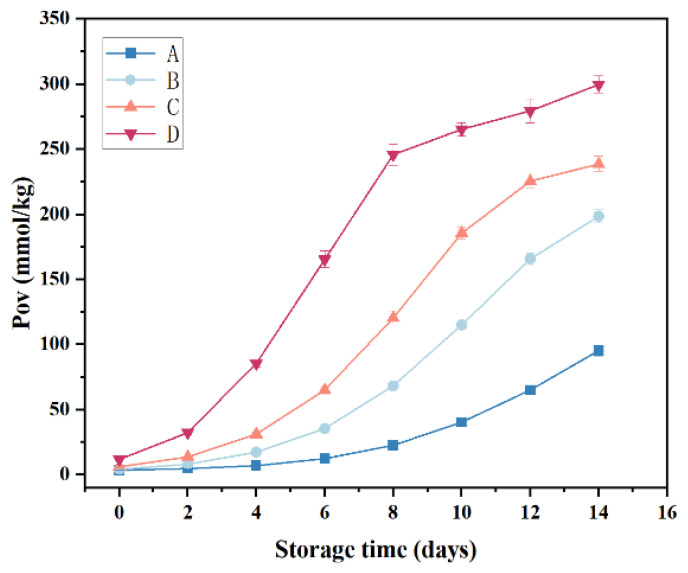
Changes in POV of YFTO obtained using different extraction processes during accelerated storage. Note: Samples were stored at 60 °C in the dark for 14 d. NETO, non-enzymatic ultrasound-assisted extraction oil; PTO, papain-assisted extraction oil; TTO, trypsin-assisted extraction oil; ATO, Alcalase-assisted extraction oil.

To compare primary oxidation development among YFTO samples obtained using different extraction process chains, POV was monitored during accelerated storage at 60 °C for 14 d (Figure 7).

As shown in Figure 7, the absolute POV increased in all groups during storage, although the samples had different day-0 values. NETO maintained the lowest absolute POV throughout storage, whereas ATO showed the highest values. The baseline-corrected changes showed the same overall ordering as the absolute POV curves (NETO < PTO < TTO < ATO). At day 14, the mean ΔPOVs of NETO, PTO, TTO, and ATO were 91.7, 193.6, 232.3, and 287.7 mmol/kg, respectively. These results indicate that the differences in oxidation development were not attributable solely to the initial POV values and that NETO showed the smallest overall accumulation of primary oxidation products under the tested conditions.

The observed differences may reflect the combined effects of the complete extraction process chains on the initial oxidation status and subsequent oxidation development of the oils. Differences in processing duration, aqueous-phase exposure, and thermal history may therefore have affected both the initial POV and its subsequent increase. Previous studies have also shown that extraction processes can influence the composition and storage stability of fish oil [41]. Lipid oxidation is affected by several factors, including fatty acid unsaturation, oxygen exposure, temperature, pro-oxidant components, and system composition, while antioxidant activity depends on the reaction system and evaluation mechanism [42,43].

The larger baseline-corrected increases observed in the enzyme-assisted oils may be associated with prolonged aqueous exposure, reaction temperature, pH conditions, emulsion formation, and the subsequent enzyme-inactivation treatment, rather than with enzymatic hydrolysis alone. This interpretation was generally consistent with the higher relative abundances of putative oxidation-related marker signals observed in these oils. It also agrees with previous reports indicating that fish oil flavor deterioration and quality changes in enzymatically extracted fish oil are closely associated with lipid oxidation [44].

Because oxidative stability was evaluated only by POV at a single accelerated-storage temperature, the results primarily reflect the accumulation of primary oxidation products under the present test conditions and cannot be used to directly predict normal shelf life. Additional secondary oxidation indices and storage tests at multiple temperatures are required for a more comprehensive assessment. Overall, under the tested conditions, NETO showed lower absolute POVs and smaller baseline-corrected increases than the selected enzyme-assisted process-chain oils.

## 4. Conclusions

This study optimized a non-enzymatic ultrasound-assisted extraction process for recovering YFTO from YFTH using oil recovery as the response variable. The validated local-optimum conditions were a nominal ultrasonic power setting of 58.3%, a heat treatment temperature of 64.0 °C, a liquid-to-solid ratio of 3.0 mL/g, and an ultrasonic treatment time of 31 min, resulting in an oil recovery of 63.92 ± 0.24%.

Under the tested conditions, NETO showed lower AV, initial POV, and p-AnV values, a lighter color, and lower relative abundances of putative oxidation-related electronic-nose marker signals than PTO, TTO, and ATO. These differences reflect comparisons among complete extraction process chains that varied in pH, processing duration, aqueous exposure, ultrasound treatment, and thermal history, and therefore cannot be attributed solely to the absence of exogenous protease. Fatty acid analysis showed no significant difference in the relative EPA proportion among the four oils. The relative DHA proportion in NETO was higher than that in ATO but did not differ significantly from those in PTO and TTO, whereas the relative ΣPUFA proportion was higher than those in TTO and ATO but not significantly different from that in PTO. Because these data were expressed as area-normalized percentages, they do not represent the absolute recovery of individual fatty acids.

During accelerated storage at 60 °C, NETO showed lower absolute POVs and a smaller baseline-corrected increase than the selected enzyme-assisted process-chain oils, indicating less accumulation of primary oxidation products under the tested conditions. Overall, NE-UAE represents a feasible process option for YFTO recovery, providing acceptable oil recovery and favorable quality characteristics within the specific conditions and comparator process chains examined. Future studies incorporating multiple quality responses, calibrated acoustic-energy measurements, confirmatory volatile analysis, and storage evaluation under a broader range of conditions would further support process optimization and practical application.

## Figures and Tables

**Table 1:** Proximate composition of YFTH.

Sample	Moisture (g/100 g)	Crude Protein (g/100 g)	Crude Lipid (g/100 g)	Ash (g/100 g)
YFTH	59.15 ± 0.51	15.32 ± 0.15	14.90 ± 0.21	8.90 ± 0.24

Note: Values are expressed as mean ± SD (*n* = 3). YFTH, yellowfin tuna head.

**Table 2:** Oil recovery of yellowfin tuna head oil obtained by different extraction processes.

Extraction Method	NETO	PTO	TTO	ATO
Oil recovery (%)	63.92 ± 0.24 ^b^	62.69 ± 0.38 ^c^	56.48 ± 0.52 ^d^	67.72 ± 0.41 ^a^

Note: Values are expressed as mean ± SD (*n* = 3). Different lowercase letters within the same row indicate significant differences among extraction processes according to Duncan’s multiple range test (*p* < 0.05). NETO, non-enzymatic ultrasound-assisted extraction oil; PTO, papain-assisted extraction oil; TTO, trypsin-assisted extraction oil; ATO, Alcalase-assisted extraction oil.

**Table 3:** Physicochemical properties of YFTO obtained using different extraction processes.

Project	NETO	PTO	TTO	ATO	Crude Fish Oil Standard ^1^	Refined Fish Oil Standard ^1^	International Standard ^2^
Moisture and volatiles /%	0.09 ± 0.03 ^a^	0.10 ± 0.07 ^a^	0.10 ± 0.17 ^a^	0.07 ± 0.05 ^a^	≤0.30	≤0.10	–
Acid value /(mg/g)	0.77 ± 0.07 ^d^	1.51 ± 0.06 ^c^	2.11 ± 0.06 ^a^	1.88 ± 0.11 ^b^	≤8.00	≤1.00	≤3.00
Peroxide value /(mmol/kg)	1.85 ± 0.13 ^d^	6.43 ± 0.16 ^c^	10.25 ± 0.16 ^b^	16.68 ± 0.19 ^a^	≤6.00	≤2.50	≤2.50
Anisidine value	1.1 ± 0.21 ^d^	5.1 ± 0.06 ^b^	4.2 ± 0.11 ^c^	6.3 ± 0.03 ^a^	–	≤20.0	≤20.0
Unsaponifiable /%	0.88 ± 0.26 ^a^	0.73 ± 0.17 ^a^	0.65 ± 0.07 ^a^	0.60 ± 0.07 ^a^	–	≤1.50	
Iodine value /(g/100 g)	184 ± 2.16 ^a^	154 ± 1.80 ^c^	164 ± 2.46 ^b^	134 ± 1.37 ^d^	≥120.00	≥140.00	
Insoluble impurities /%	0.07 ± 0.08 ^a^	0.09 ± 0.02 ^a^	0.04 ± 0.02 ^a^	0.10 ± 0.08 ^a^	–	≤0.10	

Note: Values are expressed as mean ± SD (*n* = 3). Different superscript lowercase letters indicate significant differences (*p* < 0.05). ^1^ and ^2^ indicate the limits specified in SC/T 3502–2016 and CAC Codex Stan 329–2017, respectively. “–“ indicates that no limit is specified in the corresponding standard. NETO, non-enzymatic ultrasound-assisted extraction oil; PTO, papain-assisted extraction oil; TTO, trypsin-assisted extraction oil; ATO, Alcalase-assisted extraction oil.

**Table 4:** Color parameters of YFTO obtained using different extraction processes.

Samples	L*	a*	b*	C*	h°	YI
NETO	54.8 ± 0.78 ^a^	−0.2 ± 0.06 ^d^	30.8 ± 0.48 ^c^	30.80 ± 0.19 ^d^	90.4 ± 0.48 ^a^	56.20 ± 0.81 ^c^
PTO	38.7 ± 0.85 ^b^	18.3 ± 0.02 ^c^	32.3 ± 0.75 ^c^	37.12 ± 0.56 ^c^	60.5 ± 0.62 ^b^	83.46 ± 0.51 ^b^
TTO	24.6 ±0.80 ^c^	27.9 ± 0.18 ^a^	41.8 ± 1.34 ^a^	50.26 ± 0.83 ^a^	56.3 ± 0.36 ^c^	169.92 ± 0.68 ^a^
ATO	23.1± 0.66 ^c^	26.3 ± 0.09 ^b^	39.3 ± 0.92 ^b^	47.29 ± 0.72 ^b^	56.2 ± 0.19 ^c^	170.13 ± 0.58 ^a^

Note: Values are expressed as mean ± SD (*n* = 3). Different lowercase letters within the same column indicate significant differences among extraction processes according to Duncan’s multiple range test (*p* < 0.05). L*, lightness; a*, redness/greenness; b*, yellowness/blueness; C*, chroma; h°, hue angle; YI, yellowness index. NETO, non-enzymatic ultrasound-assisted extraction oil; PTO, papain-assisted extraction oil; TTO, trypsin-assisted extraction oil; ATO, Alcalase-assisted extraction oil.

**Table 5:** Fatty acid composition of YFTO obtained by different extraction processes.

Fatty Acids	Symbol	Relative Proportions (%) NETO	Relative Proportions (%) PTO	Relative Proportions (%) TTO	Relative Proportions (%) ATO
Myristic acid	C14:0	2.61 ± 0.13 ^d^	3.34 ± 0.22 ^c^	5.92 ± 0.12 ^a^	5.49 ± 0.24 ^b^
Pentadecanoic acid	C15:0	0.64 ± 0.25 ^b^	2.11 ± 0.14 ^c^	4.76 ± 0.42 ^b^	5.89 ± 0.29 ^a^
Palmitic acid	C16:0	17.42 ± 0.75 ^c^	24.74 ± 1.22 ^a^	23.34 ± 0.61 ^a^	24.57 ± 1.52 ^a^
Margaric acid	C17:0	0.62 ± 0.04 ^b^	1.28 ± 0.32 ^b^	1.47 ± 0.12 ^ab^	1.95 ± 0.51 ^a^
Stearic acid	C18:0	4.89 ± 0.30 ^b^	6.75 ± 0.61 ^a^	6.52 ± 0.41 ^a^	6.49 ± 0.66 ^a^
Arachidic acid	C20:0	0.2 ± 0.18 ^b^	0.24 ± 0.12 ^b^	0.22 ± 0.01 ^b^	0.57 ± 0.16 ^a^
Behenic acid	C22:0	0.09 ± 0.01 ^b^	0.12 ± 0.02 ^b^	0.11 ± 0.04 ^b^	1.28 ± 0.76 ^a^
Lignoceric acid	C24:0	0.08 ± 0.01 ^b^	0.15 ± 0.02 ^b^	1.57 ± 0.12 ^a^	1.24 ± 0.36 ^a^
Total saturated fatty acids	ΣSFA	26.55 ± 1.37 ^d^	38.73 ± 1.45 ^c^	43.91 ± 2.53 ^b^	47.48 ± 1.49 ^a^
Myristoleic acid	C14:1	ND *	0.07 ± 0.04 ^c^	0.14 ± 0.07 ^b^	0.57 ± 0.06 ^a^
Palmitoleic acid	C16:1	4.12 ± 0.77 ^a^	5.24 ± 0.89 ^a^	4.59 ± 0.94 ^a^	6.05 ± 0.54 ^a^
Heptadecenoic acid	C17:1	ND *	0.64 ± 0.24 ^a^	0.57 ± 0.19 ^a^	0.51 ± 0.27 ^a^
*trans*-Oleic acid	C18:1 n9 t	0.22 ± 0.11 ^a^	0.19 ± 0.10 ^a^	0.18 ± 0.04 ^a^	0.17 ± 0.09 ^a^
*cis*-Oleic acid	C18:1 n9 c	25.62 ± 1.20 ^a^	17.02 ± 0.98 ^b^	12.93 ± 0.74 ^c^	12.15 ± 1.73 ^c^
Eicosenoic acid	C20:1	3.68 ± 0.82 ^a^	0.75 ± 0.34 ^b^	0.65 ± 0.14 ^b^	0.61 ± 0.17 ^b^
Erucic acid	C22:1	0.6 ± 0.57 ^a^	0.44 ± 0.31 ^a^	0.94 ± 0.28 ^a^	1.33 ± 0.51 ^a^
Nervonic acid	C24:1	0.76 ± 0.21 ^a^	0.19 ± 0.12 ^b^	0.15 ± 0.12 ^b^	0.13 ± 0.10 ^b^
Total monounsaturated fatty acids	ΣMUFA	35.00 ± 1.04 ^a^	24.54 ± 1.52 ^b^	20.15 ± 1.47 ^c^	21.52 ± 1.64 ^c^
Linoleic acid (*cis*)	C18:2 n6 c	1.21 ± 0.74 ^a^	1.22 ± 0.26 ^a^	1.03 ± 0.54 ^a^	0.85 ± 0.43 ^a^
*trans*-Linoleic acid	C18:2 n-6 t	0.16 ± 0.03 ^a^	0.03 ± 0.01 ^c^	0.05 ± 0.02 ^b^	0.01 ± 0.00 ^c^
Alpha-linolenic acid	C18:3 n3	0.77 ± 0.12 ^a^	0.22 ± 0.15 ^c^	0.45 ± 0.09 ^c^	0.51 ± 0.16 ^b^
Gamma-linolenic acid	C18:3 n6	0.08 ± 0.02 ^a^	0.13 ± 0.12 ^a^	0.18 ± 0.15 ^a^	0.07 ± 0.04 ^a^
Eicosadienoic acid	C20:2	0.27 ± 0.07 ^a^	0.47 ± 0.14 ^a^	0.38 ± 0.21 ^a^	0.31 ± 0.25 ^a^
Eicosatrienoic acid (n-3)	C20:3 n3	0.11 ± 0.06 ^a^	0.32 ± 0.24 ^a^	0.46 ± 0.31 ^a^	0.35 ± 0.08 ^a^
Arachidonic acid	C20:4 n6	1.23 ± 0.49 ^a^	1.74 ± 0.81 ^a^	1.41 ± 0.39 ^a^	1.2 ± 0.84 ^a^
Eicosapentaenoic acid (EPA)	C20:5 n3	7.43 ± 1.34 ^a^	5.83 ± 1.41 ^a^	5.9 ± 1.36 ^a^	5.53 ± 0.91 ^a^
Docosahexaenoic acid (DHA)	C22:6 n3	27.13 ± 1.34 ^a^	26.57 ± 2.00 ^a^	25.11 ± 1.45 ^a^	21.59 ± 2.04 ^b^
Total polyunsaturated fatty acids	ΣPUFA	38.39 ± 1.08 ^a^	36.53 ± 0.72 ^ab^	34.97 ± 1.44 ^b^	30.42 ± 1.53 ^c^
Others		0.06 ± 0.02 ^c^	0.2 ± 0.11 ^c^	0.97 ± 0.34 ^a^	0.58 ± 0.04 ^b^

Note: Values are expressed as mean ± SD (*n* = 3). Different lowercase letters within the same row indicate significant differences among extraction processes according to Duncan’s multiple range test (*p* < 0.05). * Not detected. SFA, saturated fatty acids; MUFA, monounsaturated fatty acids; PUFA, polyunsaturated fatty acids; EPA, eicosapentaenoic acid; DHA, docosahexaenoic acid. NETO, non-enzymatic ultrasound-assisted extraction oil; PTO, papain-assisted extraction oil; TTO, trypsin-assisted extraction oil; ATO, Alcalase-assisted extraction oil.

## Data Availability

The original contributions presented in this study are included in the article. Further inquiries can be directed to the corresponding author.

## Supplementary Materials

The following supporting information can be downloaded at: https://www.mdpi.com/article/10.3390/foods15142524/s1, Table S1: Coded levels and experimental values of independent variables; Table S2: Experimental design and results of response surface methodology (RSM); Table S3: Analysis of variance (ANOVA) for the quadratic response surface model of yellowfin tuna head oil (YFTO) recovery.
