# Supplementary material for: Optimization of Non-Enzymatic Ultrasound-Assisted Extraction of Yellowfin Tuna Head Oil and Comparison with Enzyme-Assisted Extraction Oils
Source: Foods. 2026 Jul 16;15(14):2524. doi: 10.3390/foods15142524 (PMC13409540; doi:10.3390/foods15142524)
Supplement: Supplementary file 1 [file foods-15-02524-s001.zip › foods-4422677-supplementary.pdf]

Table S1. Coded levels and experimental values of independent variables.

Independent variables	Coded Variable Level		
	-1	0	1
Ultrasonic power (%) X_1	40	60	80
Thermal treatment temperature (°C) X_2	55	65	75
Liquid-to-solid ratio (mL/g) X_3	2	3	4
Ultrasonic treatment time (min) X_4	20	30	40

Table S2. Experimental design and results of response surface methodology (RSM).

Run	Variable				Oil recovery (%)
	X_1 (%)	X_2 (°C)	X_3 (mL/g)	X_4 (min)	
1	0	-1	-1	0	57.64
2	1	0	0	1	58.3
3	0	0	1	1	60.45
4	-1	0	0	-1	59.15
5	0	1	0	1	54.61
6	0	-1	1	0	57.58
7	1	0	-1	0	58.1
8	0	0	0	0	64.61
9	1	0	1	0	58.4
10	-1	0	0	1	59.53
11	0	0	0	0	63.82
12	-1	0	1	0	59.17
13	0	0	0	0	64.28
14	0	0	1	-1	60.35
15	0	0	-1	1	59.81
16	-1	-1	0	0	57.13
17	0	-1	0	-1	57.35
18	1	1	0	0	53.24
19	-1	0	-1	0	59.25
20	0	-1	0	1	58.9
21	0	0	0	0	64.14
22	1	0	0	-1	57.68
23	0	1	0	-1	55.12
24	-1	1	0	0	54.89
25	0	1	1	0	54.9
26	0	1	-1	0	55.3
27	0	0	-1	-1	59.12
28	1	-1	0	0	56.42
29	0	0	0	0	64.11

Table S3. Analysis of variance (ANOVA) for the quadratic response surface model of yellowfin tuna head oil (YFTO) recovery.

**Source**	**Sum of Squares**	**df**	**Mean Squar**	**F-value**	**p-value**	
Model	271.59	14	19.40	175.47	< 0.0001	**
X_1-Ultrasonic power	4.06	1	4.06	36.72	< 0.0001	**
X_2-Temperature	23.97	1	23.97	216.81	< 0.0001	**
X_3-Liquid-to-solid ratio	0.2214	1	0.2214	2.00	0.1789	
X_4-Ultrasonic time	0.6674	1	0.6674	6.04	0.0277	*
X_1X_2	0.2209	1	0.2209	2.00	0.1793	
X_1X_3	0.0361	1	0.0361	0.3265	0.5768	
X_1X_4	0.0144	1	0.0144	0.1302	0.7236	
X_2X_3	0.0289	1	0.0289	0.2614	0.6171	
X_2X_4	1.06	1	1.06	9.60	0.0079	**
X_3X_4	0.0870	1	0.0870	0.7871	0.3900	
X_1^2	70.13	1	70.13	634.32	< 0.0001	**
X_2^2	200.56	1	200.56	1814.10	< 0.0001	**
X_3^2	31.02	1	31.02	280.58	< 0.0001	**
X_4^2	29.97	1	29.97	271.04	< 0.0001	**
Residual	1.55	14	0.1106			
Lack of Fit	1.22	10	0.1218	1.47	0.3775	
**R^2**	0.9943					
**R^2adj**	0.9887					
**C.V. (%)**	0.5661					

Note: **p < 0.01 represents statistical significance. R2 = coefficients of determination; R2adj = adjusted R2; C.V. (%) = coefficient of variation.
